# Optimizing Sleep in Athletes: The Potential of α-Lactalbumin in Nutrition Intervention

**DOI:** 10.2174/0109298665363873250623103811

**Published:** 2025-07-11

**Authors:** Jingjing Li, Xuepeng Bian

**Affiliations:** 1 Physical Education College, Shanghai University, Shanghai, China;; 2 Department of Rehabilitation, School of International Medical Technology, Shanghai Sanda University, Shanghai, China

**Keywords:** α-lactalbumin, sleep quality, athletes, neurotransmitter regulation, inflammation, oxidative stress

## Abstract

Athletes frequently encounter sleep deprivation due to the demands of high-intensity training and competition, which can significantly impair their physical recovery and athletic performance. α-Lactalbumin (α-LA), a key component of whey protein that is rich in tryptophan, has been shown to promote the synthesis of serotonin and melatonin, thereby regulating sleep cycles. Moreover, α-LA has demonstrated the ability to reduce inflammation and oxidative stress associated with fatigue and stress, further contributing to improved sleep quality. This review provides a critical evaluation of the current evidence supporting the role of α-LA in enhancing sleep quality in athletes through mechanisms such as neurotransmitter regulation, immune function improvement, and enhancement of antioxidant defenses. Additionally, it highlights the necessity for further research into the differential effects of α-LA on sleep across various sports and gender groups, as well as its potential synergistic interactions with other nutrients. These insights are essential for developing optimized nutritional interventions aimed at enhancing athletic performance.

## 1 INTRODUCTION

Sleep is an active, repetitive, and reversible state characterized by perceptual detachment from the environment and a diminished response to external stimuli. This state is essential for bodily and cerebral recovery, memory consolidation, and immune function [1]. However, athletes often encounter challenges such as reduced sleep duration, extended sleep latency, and compromised sleep quality due to factors like training-related fatigue, competitive stress, negative emotions, and jet lag [2, 3]. These sleep disturbances may elevate the risk of sports injuries and adversely affect athletic performance and competitive outcomes [4-6]. Consequently, athletes require longer and higher-quality sleep to enhance physical recovery after intense training or competition when compared to the general population [7, 8].

A balanced protein intake is fundamental for maintaining optimal physical function and significantly impacts athletic performance. A high-protein diet can reduce the number of awakenings [9], extend sleep duration [7, 10], and enhance sleep quality. However, excessive protein intake may increase sleep latency and reduce sleep quality [7, 11]. This phenomenon suggests a complex relationship between protein intake and sleep, with specific effects likely influenced by the quantity, timing, and amino acid composition of the protein consumed. Whey protein, a high-quality protein extracted from mammalian milk, is rich in branched-chain and essential amino acids, which makes it highly digestible and bioavailable. Thus, it is often called the “king of proteins”. As a component of whey protein, α-lactalbumin (α-LA) has garnered significant attention in sports science due to its role in promoting protein synthesis and muscle repair [12]. Recent studies have increasingly focused on the potential of α-LA to improve sleep quality. However, the mechanisms by which α-LA affects athletes' sleep have not been thoroughly explored or systematically investigated. Therefore, this review aims to summarize the existing research on α-LA and sleep among athletes, providing a reference and scientific basis for the application of α-LA in improving sleep quality within this population.

To this end, Web of Science, PubMed, and CNKI (China) were queried using (“α-lactalbumin” OR “whey protein”) AND athletes AND sleep. After de-duplication, title- and abstract-level screening, and full-text appraisal, six original studies that quantified sleep outcomes in athletes receiving α-lactalbumin were retained (TA 1). A parallel search for “whey protein” AND sleep identified three putative mechanisms: (i) modulation of the tryptophan (Trp)-serotonin-melatonin axis, (ii) anti-inflammatory action, and (iii) attenuation of oxidative stress. These mechanisms are detailed in the following sections.

## 2 SLEEP

### 2.1 Overview of Sleep

Mammalian sleep typically consists of two main stages: non-rapid eye movement (NREM) sleep and rapid eye movement (REM) sleep [13]. During NREM sleep, parasympathetic activity predominates, characterized by high-amplitude, low-frequency brain waves, increased gastrointestinal activity, decreased muscle tone, and slow eye movements. This stage is particularly important for the consolidation of procedural memory [14]. REM sleep, in contrast, is marked by a predominance of sympathetic activity and is characterized by low amplitude, high-frequency brain waves, increased heart rate, elevated blood pressure, muscle relaxation, and REM. Disruption of this stage can adversely affect spatial and situational memory [15].

According to statistics from the World Health Organization (WHO), approximately 27% to 45% of adults worldwide report experiencing sleep problems. These issues include insomnia, sleep apnea, REM sleep behavior disorder, circadian rhythm disorders, excessive sleepiness, and NREM sleep arousal disorders [16]. Such sleep disturbances can increase the risk of various chronic diseases, including cardiovascular, cerebrovascular, endocrine, and neuropsychiatric disorders. Therefore, improving sleep quality and management has become a critical public health priority.

### 2.2 Sleep Issues in Athletes

Previous studies have shown that athletes experience significantly reduced sleep quality compared to age- and gender-matched non-athletes [17]. Research also indicates that a substantial proportion of athletes, ranging from 50% to 78%, suffer from sleep disorders, with 22%-26% experiencing severe disturbances [18]. The primary factors contributing to sleep disorders in athletes include intensive training and competition, physical and psychological stress, environmental influences, circadian rhythm disruptions, and recovery demands. Moreover, high-intensity, high-load training can result in pronounced physical and mental fatigue, which negatively impacts sleep quality. Consequently, athletes' sleep quality tends to deteriorate as training duration, intensity, and load increase [19].

**Figure 1:**
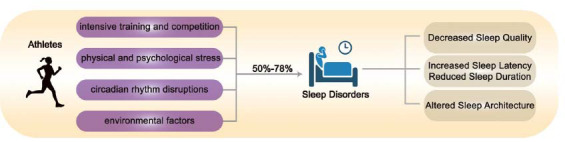
Sleep disturbances in athletes. Various factors contribute to sleep disturbances in athletes, including intensive training and competition, physical and psychological stress, circadian rhythm disruptions, and environmental factors. These factors collectively impair sleep quality, prolong sleep latency, reduce sleep duration, and alter sleep architecture.

Additionally, stress, tension, and anxiety during competition—especially in the lead-up to major events—are major contributors to sleep disturbances. These stressors can lead to prolonged sleep onset latency and reduced sleep duration [20]. Furthermore, athletes often need to adapt to changes in competition schedules and venues, which can disrupt circadian rhythms and negatively impact sleep architecture and overall quality. Due to their increased sleep requirements and higher incidence of sleep disorders, athletes must prioritize sleep quality to ensure optimal performance and overall health (Figure **1**).

## 3 EFFECTS OF α-LA SUPPLEMENTATION ON ATHLETE SLEEP

Protein intake plays a crucial role in tissue synthesis and repair [21] and supporting immune function [22]. Importantly, it is closely related to sleep regulation. Research has indicated that a diet containing 20% protein improves scores on the Pittsburgh Sleep Quality Index (PSQI) in energy-restricted overweight and obese adults compared to a diet with 10% protein [10]. However, increasing the protein content beyond this level does not yield additional benefits and may instead result in greater arousal and restlessness [23], suggesting an optimal protein intake for promoting sleep. Moreover, the timing of protein intake plays a significant role in sleep outcomes. For instance, a four-week supplementation with casein hydrolysate markedly decreased the proportion of wakefulness and increased total sleep time in mice, with these effects becoming more apparent after just two weeks [24]. Reducing the total amount or proportion of amino acids, such as tryptophan, can also affect REM sleep proportion and efficiency [25].

These findings provide evidence that multiple factors modulate the impact of protein supplementation on sleep. As a widely used supplement in recent years, whey protein improves overall physical function and regulates sleep, thereby influencing physical performance. These effects are likely mediated by its high-quality protein composition.

### 3.1 Whey Protein and α-LA

Whey protein, extracted from mammalian milk, primarily comprises α-LA, β-lactoglobulin, bovine serum albumin, immunoglobulins, and various bioactive components. Among these, α-LA is particularly noteworthy as a calcium-binding protein composed of approximately 123-125 amino acids, including cystine, lysine, and cysteine. Notably, α-LA is also the richest natural source of the essential amino acid Trp [26].

Compared to other whey proteins, α-LA offers distinct advantages. Firstly, α-LA is more easily digested and absorbed, resulting in a rapid increase in blood levels of branched-chain amino acids (BCAAs) such as leucine, isoleucine, and valine, which are beneficial for muscle protein synthesis and post-exercise recovery [12, 27]. Additionally, α-LA exhibits anti-inflammatory and antioxidant properties, primarily due to the thiol groups in cysteine, which rapidly scavenge oxidants and enhance the synthesis of glutathione (GSH) for antioxidant defense [28, 29]. Moreover, upon hydrolysis, α-LA generates immunomodulatory peptides that can activate neutrophils and enhance macrophage phagocytic activity [30-32].

Importantly, α-LA has also been shown to improve sleep quality potentially, an effect that has not been observed with other types of whey proteins [33]. These advantages position α-LA as an optimal choice for athletes seeking to expedite recovery, enhance muscle protein synthesis, bolster immune function, and improve sleep quality.

### 3.2 α-LA and Athlete Sleep

Preliminary evidence on the potential effects of α-LA on sleep suggests that evening supplementation in stress-sensitive individuals improves sleep quality, reduces sleepiness, and enhances alertness and wakefulness the following morning [34, 35]. In healthy adults, supplementation with 20 g of α-LA one hour before bedtime significantly improves total sleep time and sleep efficiency. Specifically, objective sleep duration and subjective sleep perception increase by 12.8% and 10.8%, respectively, and objective sleep efficiency improves by 7% [36].

As presented in Table **1**, the effects of α-LA supplementation on sleep quality and exercise recovery in athletes remain inconclusive. Several factors may account for these discrepancies, with intervention duration being a primary determinant. Short-term supplementation protocols (*e.g.,* 3-5 consecutive days in Studies 1, 2, and 3) generally failed to elicit significant improvements in sleep quality. In contrast, longer-term interventions (*e.g.,* the 3-week trial in Study 5) were more likely to positively affect sleep architecture and post-exercise recovery. This suggests that prolonged supplementation may facilitate the gradual accumulation of Trp and its related metabolites in plasma, enhancing sleep parameters and promoting post-exercise recovery. Another key factor influencing the effects of α-LA supplementation may be athletes' baseline protein intake. The reviewed studies included participants ranging from elite athletes to semi-professional and regularly trained individuals [37]. In studies 1 and 2, α-LA or whey protein supplementation in elite athletes did not significantly improve sleep parameters. This may be attributed to the already high-protein diets of elite athletes, resulting in a “ceiling effect,” wherein additional supplementation fails to further elevate Trp levels or modulate sleep-regulatory mechanisms. Conversely, in semi-professional or recreational athletes with pre-existing sleep disturbances (Studies 5 and 6), α-LA supplementation appeared to exert a more pronounced effect, improving sleep architecture and enhancing next-morning cognitive performance.

Although most studies administered α-LA approximately two hours before bedtime (except Study 2), aiming to optimize the plasma Trp to large neutral amino acids (LNAA) ratio at sleep onset, variations in dosage and composition may also influence outcomes. Intakes across studies ranged from 40 to 60 grams, and differences in Trp content and its ratio to other essential amino acids may have affected neurotransmitter metabolism and the timing of synthesis. These variations may contribute to inconsistencies in the effectiveness of α-LA supplementation on sleep and exercise recovery [38].

Overall, the duration of intervention, timing of supplementation, athlete characteristics, and choice of sleep monitoring method may be more important determinants of α-LA’s impact on sleep and motor recovery. At the same time, age does not seem to be a significant factor, but this may be due to the limited sample size.

Notably, although α-LA supplementation has shown positive effects on improving sleep in some studies involving athletes, the results remain inconsistent. Consequently, further randomized controlled trials are necessary to clarify its impact. Additionally, current research on the influence of α-LA supplementation on athletes' sleep often overlooks alterations in sleep architecture, which may differentially impact athletic performance. Furthermore, existing research has yet to comprehensively investigate the mechanisms through which α-LA supplementation enhances sleep in athletes [39-41].

## 4 MOLECULAR MECHANISMS OF α-LA IN SLEEP IMPROVEMENT

### 4.1 Regulation of Trp, Serotonin (5-Hydroxytryptamine, 5-HT), and Melatonin Levels

α-LA is abundant in essential amino acids, particularly Trp [26]. Investigations into the sleep-regulating properties of α-LA originate from early research on the relationship between Trp and sleep. Initial studies on infant formula demonstrated that supplementation with adequate amounts of Trp or its precursors significantly improved infant sleep patterns [42]. Furthermore, a Trp-enriched protein diet has been shown to effectively restore sleep following food deprivation in rats [43]. These findings provide a foundational biochemical basis for the potential role of α-LA in sleep modulation.

It has been reported that supplementation with Trp could reduce wakefulness after sleep onset, with more pronounced effects observed at doses equal to or exceeding 1g [44]. Furthermore, Trp serves as a precursor for the synthesis of 5-HT [45], which is a critical precursor for melatonin synthesis. As both 5-HT and melatonin are pivotal regulators of the sleep-wake cycle [46], thus, Trp supplementation can significantly improve sleep quality and enhance the synchronization of circadian rhythms by increasing the levels of 5-HT and melatonin [25]. Therefore, elucidating the roles of 5-HT and melatonin in sleep regulation is crucial for understanding the relationship between α-LA supplementation and sleep enhancement in athletes.

#### 4.1.1 5-HT

5-HT is primarily synthesized in the enterochromaffin cells of the gut and the dorsal raphe nucleus of the brain, with the latter and its projection areas integral to arousal pathways [47]. Studies have demonstrated that supplementation with 5-hydroxytryptophan (5-HTP), a direct precursor of 5-HT, can improve sleep quality while increasing serum 5-HT levels. In individuals with sleep disorders, 12 weeks of 5-HTP supplementation significantly improved subjective sleep scores, accompanied by enhanced gut microbiota diversity and abundance [48]. Notably, specific gut microbial strains, including Lactobacillus, Lactobacillus rhamnosus, and Bifidobacterium, facilitate sleep regulation by synthesizing 5-HT and γ-aminobutyric acid (GABA), providing mechanistic insights into the microbiome’s role in sleep modulation [49, 50]. Conversely, sleep deprivation can deplete beneficial gut flora and exacerbate dysbiosis, further underscoring the bidirectional relationship between sleep and the gut microbiome [49].

Since 5-HT cannot cross the blood-brain barrier, its synthesis in the brain depends on the availability of Trp at the site of synthesis [51]. The transport of circulating Trp into the brain is facilitated by LNAA carriers [52]. Under normal conditions, these carriers are saturated and compete to transport BCAAs (such as leucine, isoleucine, and valine), phenylalanine, tyrosine, and Trp [52]. Consequently, brain Trp levels depend on the absolute concentration of Trp in the blood and the Trp/LNAA ratio [26]. Furthermore, studies have shown that a significant increase in brain Trp content and 5-HT release requires a ≥40-50% increase in the peripheral Trp/LNAA ratio [26, 34]. Compared to other proteins, α-LA has a high Trp content and the highest Trp/LNAA ratio [26]. Additionally, the intake of α-LA can increase the plasma Trp/LNAA ratio between 16% and 130% [34, 35, 53, 54]. This significant increase in the Trp/LNAA ratio enhances the availability of Trp in the brain, thereby boosting brain levels of both Trp and 5-HT [35].

The regulation of sleep by 5-HT primarily depends on its receptors. Among them, activation of 5-HT1A and 5-HT2C receptors can reduce anxiety and depressive symptoms, decrease sleep latency, and improve sleep quality by enhancing gastrointestinal health and other physiological states [55, 56]. Similarly, activation of the 5-HT7 receptor regulates circadian rhythms, thereby supporting a consistent sleep-wake cycle [57, 58]. Additionally, 5-HT interacts with the dopamine system to balance neurotransmitters, further promoting sleep [59]. Though 5-HT might inhibit certain sleep stages through 5-HT1A, 5-HT1B, 5-HT2A, and 5-HT6 receptors [60-64], its overall impact is considered beneficial for improving sleep quality.

Notably, the effects of α-LA may be influenced by endogenous 5-HT levels. In highly stressed individuals, baseline Trp levels are typically low, leading to reduced 5-HT status. α-LA intake can restore 5-HT to optimal levels, positively influencing cognitive and behavioral processes [53]. Conversely, the benefits are not significant in healthy individuals [54]. Similarly, athletes often experience high stress due to competitions and intense training. Therefore, α-LA supplementation might help increase Trp and 5-HT levels, thereby improving sleep quality in athletes.

#### 4.1.2 Melatonin

α-LA supplementation is beneficial for increasing circulating levels of Trp and 5-HT, which are important precursors for melatonin synthesis [65]. Melatonin, primarily synthesized by the pineal gland in the brain, is a hormone closely associated with the sleep-wake cycle. It regulates the biological clock, reducing sleep latency and the number of awakenings during the night [66]. Additionally, it increases total sleep time and improves sleep efficiency [66]. Studies have shown that melatonin supplementation can improve balance and physical performance in athletes in college after sleep deprivation [67]. Following intense late-night exercise, a 10 mg melatonin supplement can enhance total sleep time, sleep efficiency, NREM N3, and REM sleep in adolescents while reducing sleep latency, nighttime awakenings, and the duration of NREM N1 and N2 sleep [68]. Additionally, melatonin supplementation improves the antioxidant status of athletes during high-intensity exercise, reducing oxidative stress and inflammatory markers [69].

Melatonin primarily regulates sleep through melatonin receptors 1 (MT1) and MT2, with this modulation mediated by the subtypes of these receptors [70]. The MT1 subtype significantly influences REM sleep, as evidenced by a marked reduction in REM sleep when it is knocked out [71, 72]. Conversely, the MT2 subtype predominantly affects NREM sleep, with its activation significantly reducing the latency to the first NREM sleep episode and increasing NREM sleep duration without impacting the REM sleep [73, 74]. In addition to melatonin receptors, regulating sleep by melatonin is also associated with GABA. GABA, an inhibitory neurotransmitter, positively correlates with sleep duration [75]. Significantly, melatonin could enhance GABA synthesis and release by binding to and activating GABA synthase, which acts on GABA receptors to reduce sleep latency [76, 77], extend sleep duration, and improve sleep quality. Furthermore, research has also demonstrated that the activation of GABAergic neurons in mice's ventral tegmental area (VTA) can induce prolonged NREM sleep, whereas damage to these neurons increases wakefulness, with this heightened wakefulness lasting for at least four months [78].

Therefore, increasing melatonin levels through α-LA supplementation could be an effective strategy to improve sleep quality in athletes. Furthermore, the changes and interactions in melatonin receptors and the GABA system induced by α-LA supplementation and their importance in sleep regulation merit deeper investigation.

#### 4.1.3 Complementary Roles of 5-HT and Melatonin in Sleep Regulation

Trp serves as the common precursor for both 5-HT and melatonin synthesis. The initial step in this biosynthetic pathway involves the hydroxylation of Trp to 5-HTP by tryptophan hydroxylase (TPH), followed by the decarboxylation of 5-HTP to 5-HT, catalyzed by aromatic amino acid decarboxylase. 5-HT is converted into melatonin in the pineal gland through sequential N-acetylation and methylation reactions [45]. Notably, TPH functions as the rate-limiting enzyme in this pathway, and its enzymatic activity critically regulates the production of both 5-HT and melatonin, establishing a tightly interconnected biosynthetic network [79]. A study based on traditional Chinese medicine found that Shen Yuan extract reduces sleep latency and extends sleep duration in normal mice, while also counteracting sleep loss induced by chronic restraint stress. The extract elevates cortical Trp levels and upregulates TPH2 expression, enhancing 5-HT synthesis. In addition, it lowers serum corticosterone while increasing the expression of melatonin, its receptor (MT2), and cryptochrome 1 (Cry1), collectively contributing to improved sleep disturbances [80]. Conversely, melatonin supplementation boosts type 1 TPH (TPH1) activity in the pineal gland of aged rats, aiding in the restoration of pineal function and preventing the nocturnal decline of 5-HT and melatonin synthesis, ultimately establishing a positive self-regulatory loop [81].

Despite their structural continuity and shared biosynthetic origin, 5-HT and melatonin exert distinct yet complementary physiological roles in sleep regulation. 5-HT primarily facilitates slow-wave sleep, contributing to deeper and more restorative sleep phases [82], whereas melatonin plays a pivotal role in modulating circadian rhythms, particularly in initiating sleep onset and maintaining REM sleep [46]. Sleep deprivation or circadian rhythm disruption can shift Trp metabolism towards the kynurenine pathway, reducing 5-HT and melatonin synthesis and ultimately impairing sleep architecture [83, 84]. This dynamic interplay and bidirectional regulatory mechanism exemplify the intricate cross-talk within the Trp metabolic network.

### 4.2 α-LA Modulates Inflammation and Oxidative Stress

α-LA exhibits immunomodulatory properties [26] and mitigates oxidative stress by enhancing antioxidant defenses [85]. Studies have shown that sleep deprivation elevates oxidative stress and inflammatory markers, which are closely linked to diminished sleep quality [86, 87]. Conversely, increased oxidative stress and inflammation may further impair sleep quality and overall health, establishing a vicious cycle [88]. Thus, this section aims to explore the potential mechanisms by which α-LA regulates immune functions and oxidative stress and to evaluate its effects on sleep quality.

#### 4.2.1 α-LA Enhances Immune Function

Sleep deprivation significantly impacts immune responses. Specifically, four days without sleep can lead to a rapid accumulation of pro-inflammatory cytokines such as interleukin 6/17A (IL-6/17A) and chemokines (such as CXCL1 and CXCL2) in mouse blood, resulting in the neutrophil proliferation [87]. Extending this period to seven days activates mouse hippocampal microglia and astrocytes, downregulates nicotinic acetylcholine receptors, and diminishes PI3K/Akt/GSK-3β pathway activation. This alteration results in increased pro-inflammatory cytokines and decreased levels of anti-inflammatory cytokines, such as nuclear factor E2-related factor and the antioxidant enzyme heme oxygenase-1 [89]. Prolonged sleep deprivation exacerbates these effects, further disrupting metabolism and increasing the secretion of inflammatory markers such as C-reactive protein, IL-6, and tumor necrosis factor α (TNF-α) [90]. Furthermore, these inflammatory responses provoke changes in sleep architecture such as shortened overall sleep duration, sleep fragmentation, and increased REM sleep while simultaneously reducing slow-wave sleep [91]. For instance, elevated levels of IL-6 and TNF-α are associated with increased sleep fragmentation and nocturnal awakenings [92]. At the same time, IL-1 promotes NREM sleep, whereas anti-inflammatory cytokines like IL-4 and IL-10 counteract these inflammatory responses, enhancing sleep quality.

α-LA can influence inflammation through various mechanisms [93]. First, α-LA can inhibit the expression of pro-inflammatory cytokines such as TNF-α and IL-6 and block inflammatory signaling pathways, including c-Jun N-terminal kinase (JNK) and nuclear factor kappa-B (NF-κB), thereby reducing the release of inflammatory mediators [94, 95]. NF-κB, as a critical transcription factor, activates the expression of various inflammatory genes such as inducible nitric oxide synthase (iNOS) and cyclooxygenase-2 (COX-2), which increases the release of pro-inflammatory cytokines and exacerbates inflammatory responses, ultimately reducing NREM sleep [90]. Secondly, α-LA's impact on gut health also contributes to its anti-inflammatory effects. The α-LA peptide Asp-Gln-Trp can improve gut barrier function, increase the proportion of beneficial bacteria such as lactobacilli and bifidobacteria, and reduce the leakage of gut toxins like lipopolysaccharides (LPS), thereby lowering systemic inflammation [96]. These combined mechanisms underscore the potential of α-LA in modulating immune function and reducing oxidative stress, which may contribute to enhanced sleep quality.

Additionally, α-LA can reduce the production of prostaglandin E2 (PGE2) by inhibiting the activities of COX-2 and phospholipase A2 (PLA2), thereby alleviating PGE2-induced fever, pain, and sleep quality deterioration [97]. Moreover, α-LA increases circulating adiponectin levels, which has anti-inflammatory and insulin-sensitizing effects and negatively correlates with inflammatory responses [98]. α-LA also improves sleep quality by promoting the production of protective substances. For instance, it stimulates the synthesis and secretion of gastric mucin, enhancing the protective capacity of the gastric mucosa and reducing inflammation and damage to the gastric lining. This effect alleviates nighttime awakenings caused by gastric discomfort, improving sleep quality [99].

Although there are no current reports of α-LA directly regulating sleep by altering inflammatory cytokine levels, the studies above suggest that α-LA may influence sleep through its anti-inflammatory effects. However, the impact of α-LA supplementation on different sleep stages, such as NREM and REM sleep, and their correlation with inflammatory responses still requires further investigation and clarification.

#### 4.2.2 α-LA Enhances Antioxidant Capacity

During high-intensity exercise and competition, the generation of free radicals increases, leading to elevated oxidative stress and reduced sleep quality [100]. Insufficient sleep or sleep disorders can exacerbate oxidation and nitration reactions, triggering peripheral immune activation and neuroinflammation and thereby creating a vicious cycle [73]. Antioxidant supplementation with luteolin can improve cognitive decline caused by sleep deprivation [101], and a diet rich in vitamin C can also ameliorate the sleep disturbances caused by exposure to nighttime traffic noise [102]. These studies indicate that maintaining the body's redox balance is crucial for enhancing sleep quality and minimizing sleep disturbances [100].

α-LA exhibits potent effects in mitigating oxidative stress, primarily by enhancing lipid metabolism and boosting the activity of antioxidant enzymes. Specifically, α-LA hydrolysates enhance hepatic lipid degradation and reduce oxidative stress by activating the PPARα pathway in HepG2 cells treated with free fatty acids and in mice with high-fat diet-induced non-alcoholic fatty liver disease [103, 104]. These hydrolysates also decrease oxidative stress markers such as malondialdehyde and reactive oxygen species (ROS), and reduce blood pressure by inhibiting the angiotensin-converting enzyme. They also increase the activities of antioxidant enzymes such as superoxide dismutase (SOD) and glutathione peroxidase (GPx) [85, 105]. Furthermore, bioactive peptides in whey protein pancreatic hydrolysates enhance the activities of antioxidants like SOD and catalase (CAT), raise intracellular GSH levels, and promote cell proliferation while scavenging ROS [106]. Additionally, the abundant cysteine and methionine in α-LA can be converted into reduced GSH, whose thiol groups donate electrons to free radicals, thereby stabilizing their structure [29, 107, 108] and mitigating oxidative damage.

For elite middle and long-distance runners, α-LA supplementation during the specialized training phase before competition significantly increases blood levels of SOD, GPx, and glutathione-S-transferase (GST) while markedly lowering malondialdehyde levels [109]. Similarly, α-LA reduced glutathione levels and increased total antioxidant capacity and protein carbonyls in the muscle of male soccer players after speed-endurance training [110]. These findings suggest that α-LA enhances the activity of antioxidant enzymes, particularly those associated with GSH, thereby boosting the body's antioxidant capacity.

While α-LA significantly enhances the body's antioxidant defenses and improves sleep, direct evidence of its role in modulating sleep through oxidative stress remains scant. Recent studies indicate that α-LA might mitigate age-associated inflammation and oxidative stress by modulating the expression of sirtuin 1 (Sirt1) [111]. This pathway may represent a crucial mechanism by which α-LA lowers oxidative stress to improve sleep outcomes.

### 4.3 Other Potential Effects

α-LA inhibits the transport of competitive amino acids—such as tyrosine, phenylalanine, and BCAAs—thereby facilitating a more significant influx of Trp across the blood-brain barrier. This mechanism enhances the synthesis of 5-HT and melatonin in the brain [112]. Furthermore, α-LA promotes neural inhibition *via* the GABA pathway by supplying glutamine, a precursor for GABA synthesis, modulating GABA receptor activity, reducing nocturnal awakenings, and improving sleep stability [26, 113].

Additionally, α-LA augments sleep drive by promoting the accumulation of adenosine. Adenosine, which gradually builds up during wakefulness [114], is increased through ATP consumption facilitated by α-LA. This accumulated adenosine acts on A1 and A2A receptors to further inhibit arousal-promoting neurons [115], enhancing sleep depth. Concurrently, α-LA attenuates the release of norepinephrine from the locus coeruleus *via* 5-HT-mediated negative feedback, reducing arousal signals [116]. It also suppresses dopamine, orexin, and cortisol levels, which collectively help alleviate anxiety-related insomnia and improve overall sleep quality [116].

Moreover, some studies have shown that the wakefulness period of the organism is prolonged after partial damage to cholinergic cells through the injection of immunotoxins [117]. However, it may lead to an increase in REM sleep when acetylcholinesterase inhibitors are used alone. Although there are no reports yet on the direct impact of α-LA supplementation on the acetylcholine system, animal studies have shown that mice on a Trp-restricted diet exhibit significant reductions in body and brain weight, impairments in cholinergic systems and serotonergic neurons, and short-term learning and memory dysfunctions [118], suggesting that Trp intake might influence the acetylcholine system. Consequently, it is hypothesized that α-LA could potentially enhance cholinergic neurotransmission by increasing brain Trp levels, thereby affecting serotonergic neurons and leading to changes in sleep-related parameters.

## CONCLUSION AND FUTURE PERSPECTIVES

α-LA, rich in Trp and other amino acids, may enhance sleep in athletes *via* multiple mechanisms. These include regulating 5-HT and melatonin synthesis, augmenting anti-inflammatory and antioxidant capacities, and modulating multiple physiological systems. This study comprehensively summarizes and analyzes the mechanisms by which α-LA influences sleep in athletes, revealing its significant potential to improve sleep quality.

While moderate doses of α-LA are generally considered safe, prolonged high-dose usage may pose risks. Trp, a key component of α-LA, can be metabolized into kynurenine, which may be further metabolized into compounds such as quinolinic acid and 3-hydroxykynurenine. These metabolites can induce excitotoxicity by activating NMDA receptors, thereby contributing to pathological processes associated with neurodegenerative diseases and depression [119]. Therefore, developing personalized precision nutrition interventions based on nutrigenomics, metabolomics, gut microbiome, and lifestyle data is critically important for optimizing related research protocols [120]. The underlying mechanism involves individual genetic variations—such as those in the FTO and MC4R genes—that affect the metabolism and absorption of specific nutrients, thereby influencing disease risk [121]. Moreover, variations in blood biomarkers can predict individual dietary responses and help tailor nutritional interventions [122]. In addition, the composition of the gut microbiome plays a vital role in nutrient absorption, energy metabolism, and immune regulation, with inter-individual differences potentially impacting disease development [123]. Notably, the metabolism, absorption, and subsequent products of α-LA are closely linked to the gut microbiota, underscoring the need for further exploration of these underlying mechanisms using a precision nutrition approach.

Current research predominantly focuses on athletes without overt sleep disturbances, suggesting that future studies should target those experiencing sleep restriction. It is essential to consider the sleep stage characteristics of athletes to determine the optimal dosage and timing of α-LA supplementation. Existing studies have utilized a dosage range of 20-40 grams; however, the optimal dose remains uncertain. Future investigations should employ dose-gradient designs (*e.g.,* low, medium, and high dose groups) to elucidate the dose-response relationship. Regarding administration strategies, the selection of α-LA dosage should be informed by its metabolic profile, ensuring that it sufficiently elevates blood Trp levels without incurring adverse effects. Moreover, different timing strategies—such as bedtime-only *versus* divided daily intake—warrant exploration. Although current applications of α-LA are largely concentrated in endurance sports such as football, cycling, and rugby, its potential use in skill-dominated sports represents an intriguing avenue for future research.

Finally, synergistic effects between α-LA and other nutrients should be investigated to develop targeted supplementation and nutritional programs. Such integrated strategies may facilitate optimal rest and recovery during training and competition, ultimately enhancing athletic performance and overall health.

## Figures and Tables

**Table 1:** The impact of α-LA (whey protein) supplementation on sleep quality in athletes.

**Study ID**	**Exercise Type**	**Sample Size**	**Gender**	**α-LA Supplementation**			Measurement Tools*	**Sleep Changes^&^**	**References**
**Dosage**	**Timing**	**Duration**
1	Cycling	6	Male	40 g	2 h before sleep	3 d	Accelerometer, Sleep Diary, KSS	—	[39]
2	Soccer	15	Male	55 g	3 h before sleep	5 d	Arm Activity Monitor, Sleep Diary	—	[40]
3	Endurance (HIIT)	11	5 Male/6 Female	20 g	after exercise	3 d	Accelerometer, TQRS, KSS, Sleep Diary	—	[12]
				40 g	2 h before sleep				
4	Mixed-sport**^#^**	16	Female	40 g	before sleep	Single	PSG	NREM 2% ↑	[27]
5	Rugby	18	Female	40 g	2 h before sleep	3 w	Arm Activity Monitor, Sleep Diary	SOL (bye week /away game) ↓ SE (bye week) ↑	[33]
6	Athletic Training	19	8 Male/11 Female	40 g	2 h before sleep	3 d	Dry Electroencephalo-graphy, ASSQ, PSQI	NREM 2 ↑ REM↓	[41]

**Note:** *Measurement Tools: Subjective assessments included self-reported instruments such as the Pittsburgh Sleep Quality Index (PSQI), Total Quality Recovery Scale (TQRS), Karolinska Sleepiness Scale (KSS), Athlete Sleep Screening Questionnaire (ASSQ), and sleep diaries. Objective assessments comprised accelerometry, polysomnography (PSG), arm activity monitors, dry electroencephalography, and other similar passive monitoring tools. ^&^Sleep Changes: “↑” indicates an increase or improvement, “↓” indicates a decrease or deterioration, and “-” denotes no significant change. Among these, total sleep time, sleep efficiency, sleep onset latency, number of awakenings, and sleep fragmentation index were commonly reported but did not show significant differences across conditions in several studies. ^#^Mixed-sport: Participants had athletic backgrounds in football, rugby, field hockey, middle- and long-distance running, and strength training and movement.

## LIST OF ABBREVIATIONS

5-HT: 5-hydroxytryptamine
5-HTP: 5-hydroxytryptophan
α-LA: α-Lactalbumin
ASSQ: Athlete Sleep Screening Questionnaire
BCAAs: Branched-chain Amino Acids
CAT: Catalase
COX2: Cyclooxygenase-2
GABA: γ-aminobutyric Acid
GPx: Glutathione Peroxidase
GSH: Glutathione
GST: Glutathione-S-transferase
IL-6/17A: Interleukin 6/17A
iNOS: Inducible Nitric Oxide Synthase
JNK: c-Jun N-terminal Kinase
KSS: Karolinska Sleepiness Scale
LNAA: Large Neutral Amino Acids
LPS: Lipopolysaccharides
MT1: Melatonin Receptors 1
NF-κB: Nuclear Factor Kappa-B
NREM: Non-rapid Eye Movement
PGE2: Prostaglandin E2
PSQI: Pittsburgh Sleep Quality Index
PSG: Polysomnography
REM: Rapid Eye Movement
ROS: Reactive Oxygen Species
Sirt1: Sirtuin 1
SOD: superoxide Dismutase
TNF-α: Tumor Necrosis Factor α
TPH: Tryptophan Hydroxylase
TQRS: Total Quality Recovery Scale
Trp: Tryptophan
VTA: Ventral Tegmental Area
